# Multiple cutaneous nerve sheath tumours with myxoid differentiation in farmed Russian sturgeons (*Acipenser gueldenstaedtii*, Brandt and Ratzeburg 1833*)*

**DOI:** 10.1007/s11259-025-10662-7

**Published:** 2025-02-01

**Authors:** Luciana Mandrioli, Ginevra Brocca, Samuele Zamparo, Massimo Orioles, Maria Morini, Luana Cortinovis, Eleonora Fiocchi, Maral Anjomanibenisi, Anna Toffan, Tobia Pretto, Ranieri Verin

**Affiliations:** 1 Department of Veterinary Medical Sciences, Alma Mater Studiorum University of Bologna, Bologna, Italy; 2 Aquatic Diagnostic Services, Atlantic Veterinary College, University of Prince Edward Island, Charlottetown, Canada; 3 Azienda agricola troticoltura Erede Rossi Silvio, Sefro, Macerata, Italy; 4 AULSS2 Marca Trevigiana, Treviso, 31100 Italy; 5 Istituto Zooprofilattico Sperimentale delle Venezie, Padova, Italy; 6 Department of Comparative Biomedicine and Food Science (BCA), University of Padova, Padua, Italy

**Keywords:** *Acipenser gueldenstaedtii*, Cutaneous tumours, Fish neoplasms, Russian sturgeon

## Abstract

Sturgeon species are well-suited for aquaculture because of their favourable characteristics, including robustness, suitability for farming in facilities unsuitable for other fish species, and adaptability to diverse farming conditions. The Russian sturgeon (*Acipenser gueldenstaedtii*, Brandt and Ratzeburg 1833) is one of the most prominent farmed species; however, like other aquaculture species, it is susceptible to significant losses from bacterial and viral diseases. Beyond infectious causes, there are few reports documenting conditions that produce cutaneous masses in Russian sturgeons. This study presents a multidisciplinary investigation of six farmed Russian sturgeons exhibiting discrete, multiple cutaneous masses. Bacteriological analysis of tissue samples revealed the presence of *Morganella morganii* and *Aeromonas veronii* biovar *sobria*, identified as opportunistic bacteria. Virological assays targeting the principal viruses affecting sturgeon, Acipenser iridovirus and Acipenser herpesvirus, yielded negative results. Ultrastructural analysis with direct negative staining revealed no evidence of biological agents. Histologically, the dermal masses were well-demarcated, expansile, and moderately cellular, consisting of spindle-to-stellate neoplastic cells that were multifocally periodic acid–Schiff-positive and embedded in abundant alcianophilic ground substance. Immunohistochemistry with the S-100 antibody confirmed cytoplasmic staining of the neoplastic cells. A final diagnosis of cutaneous nerve sheath tumour with myxoid differentiation was made, replicating findings from a similar tumour in rainbow trout. To the best of our knowledge, this represents the first description of multiple cutaneous nerve sheath tumours in sturgeon species. The potential factors contributing to the development of this neoplastic condition are discussed.

## Introduction

Russian sturgeons belong to the Acipenseridae family and are native to the Caspian, Azov, and Black Seas. In global sturgeon aquaculture, Russian sturgeon (*Acipenser gueldenstaedtii*, Brandt and Ratzeburg 1833) farming accounts for a significant proportion because of the high quality of its caviar. The Osetra caviar produced from aquaculture represented 20% of global caviar production in 2016 (Ehletawi et al. 2020).

Various techniques and breeding systems are employed in sturgeon aquaculture worldwide. The predominant systems include flow-through systems (FT), recirculating aquaculture systems (RAS), cages, hybrid FT/RAS systems, and ponds. Other rearing methods combine systems, such as hybrid FT/ponds, cage/ponds, and RAS/ponds (Bronzi et al. 2019).

Given their adaptability to various farming methods and tolerance to low oxygen levels, sturgeon species are experiencing global growth in aquaculture. These species hold significant potential for aquaculture because of their exceptional qualities, including their adaptability to farming environments, valuable caviar, and high-quality flesh (Vilkova et al. 2022). However, as with other fish species, infectious diseases are a major limiting factor in sturgeon farming. Viral infections, in particular, cause considerable damage to the industry (Mugetti et al. 2020). Additionally, disease control in sturgeon farming is challenging because of limited knowledge of disease epidemiology and control methods (Ciulli et al. 2016). With respect to diseases involving the formation of masses, tumour-like cutaneous and oral masses consistent with mycobacterial granulomas have been reported in Russian sturgeons (Antuofermo et al. 2014).

The current case report describes the investigation of six adult Russian sturgeons presenting with multiple cutaneous masses.

## Methodology

### Animals

The sturgeons were reared on an Italian commercial farm that sourced water from a spring and maintained a constant temperature of 15 °C throughout the year. The site was previously a trout grow-out raceway facility but was converted to sturgeon production because of non-optimal temperatures for trout farming. The facility continuously monitored dissolved oxygen and temperature parameters and employed an automatic system for adding oxygen to the water, ensuring the concentration never fell below 60% saturation.

Six adult sturgeons from the runt group, weighing 3 to 6 kg, exhibited several cutaneous masses on the pectoral and anal fins as well as the barbels. Some of the masses had a cystic appearance, and they significantly impaired swimming and feeding ability. Euthanasia using an overdose of 2-phenoxyethanol (200 ppm) was performed because of the progressive growth of the masses, and a diagnostic work-up was initiated.

### Bacteriological investigations

Cutaneous masses were sampled using a loop and streaked onto Columbia Blood Agar (BA; Oxoid, Basingstoke, UK). The plates were incubated at 22 °C for 5 days, and observed daily. Isolated colonies were subcultured on BA and incubated at 22 °C for 24 h to obtain pure cultures. Bacterial identification at species level were obtained through biochemical and proteomic analyses (MALDI-TOF MS, Brucker Microflex LT, Brucker Daltonics- library MBT 8468, 2019).

Samples of core tissue from cutaneous masses (*n* = 4) were collected to investigate the presence of bacteria of the genus *Mycobacterium*.

Total DNA extraction was performed using a QIAamp DNA Mini Kit (Qiagen) according to the manufacturer’s protocol. The extracted DNA was then tested for the presence of *Mycobacterium* sp. using the end-point PCR assay (Telenti et al.^1993^).

### Virological analysis

For molecular investigation, total nucleic acids were extracted from each tumour individually (*n* = 4) using the QIAsymphony DSP Virus/Pathogen Midi Kit (Qiagen, Hilden, Germany) in combination with the automated QIAsymphony SP system (Qiagen). The extracted nucleic acids were subjected to real-time polymerase chain reaction (RT-PCR) for Acipenser iridovirus-European (AcIV-E) according to the protocol developed by Bigarrè et al. (2017) and to end-point PCR for Acipenser herpesvirus (AcHV) detection following the protocol described by Hanson et al. (2006).

Viral cultivation was carried out using the white sturgeon skin (WSSK-1) cell line (Hedrick et al. 1991). Briefly, tissue samples were ground with sterile quartz sand in sterile cooled mortars, diluted 1:10 in L-15 medium supplemented with 10% foetal bovine serum and 1% antibiotic/antimycotic solution, clarified at 4000 × *g* at 4 °C for 15 min, and incubated overnight at 4 °C. The following day, the supernatants were inoculated onto 24-hour-old WSSK-1 cells and incubated at 20 °C. Cultivation was performed for 7 days, followed by two blind passages.

### Transmission electron microscopy

A volume of the supernatant obtained from the mass homogenate as previously described for virus isolation (*n* = 4), as well as the fluid harvested from the cystic tissue, was frozen at − 20 °C, thawed in a water bath at 37 °C, and then centrifuged for 15 min at 2200 × *g*. In total, 100 µL of the supernatants were transferred into a polyallomer tube containing a Formvar-coated copper grid (200 mesh) (Electron Microscopy Science, Hatfield, UK) and centrifuged at 95,000 × *g* for 15 min using an ultracentrifuge (Airfuge Beckman, Milan, Italy). The copper grid was stained with a drop of 2% phosphotungstic acid for a few seconds. The grid was then directly observed using a Philips EM208S transmission electron microscope (Philips, Amsterdam, Netherlands) operating at 80 kV.

### Pathological investigations

Samples from the six fish masses were fixed in 10% neutral-buffered formalin, processed according to standard procedures, embedded in paraffin, and cut into 3-µm sections. The sections were stained with haematoxylin and eosin, periodic acid–Schiff (PAS), and Alcian blue pH 2.5. Immunohistochemistry was performed using an automatic immunostainer (Ventana Benchmark GX; Roche Diagnostics, Basel, Switzerland) with a diaminobenzidine detection kit (ultraView Universal DAB Kit; Roche Diagnostics). A rabbit polyclonal antibody (Z0311, Dako Omnis; Agilent Technologies, Santa Clara, CA, USA) against S-100 was applied at a dilution of 1:1,000 in phosphate-buffered saline, following the methods described in an oncologic case report of rainbow trout (Brocca et al. 2021). A brain section of a juvenile Russian sturgeon was used as positive control, and the negative control was obtained through the omission of the primary antibody on the same tissue.

## Results

Concerning bacteriology, presence of *Morganella morganii* and *Aeromonas veronii* biovar *sobria* was observed in the cutaneous masses. Molecular analysis for *Mycobacterium* presence tested negative. Regarding virology, RT-PCR for AcIV-E and AcHV, as well as isolation on cell cultures, yielded negative results. Transmission electron microscopy (TEM) also revealed no evidence of biological agents, collectively reducing the likelihood of an association between biological agents and the cutaneous masses.

**Fig. 1:**
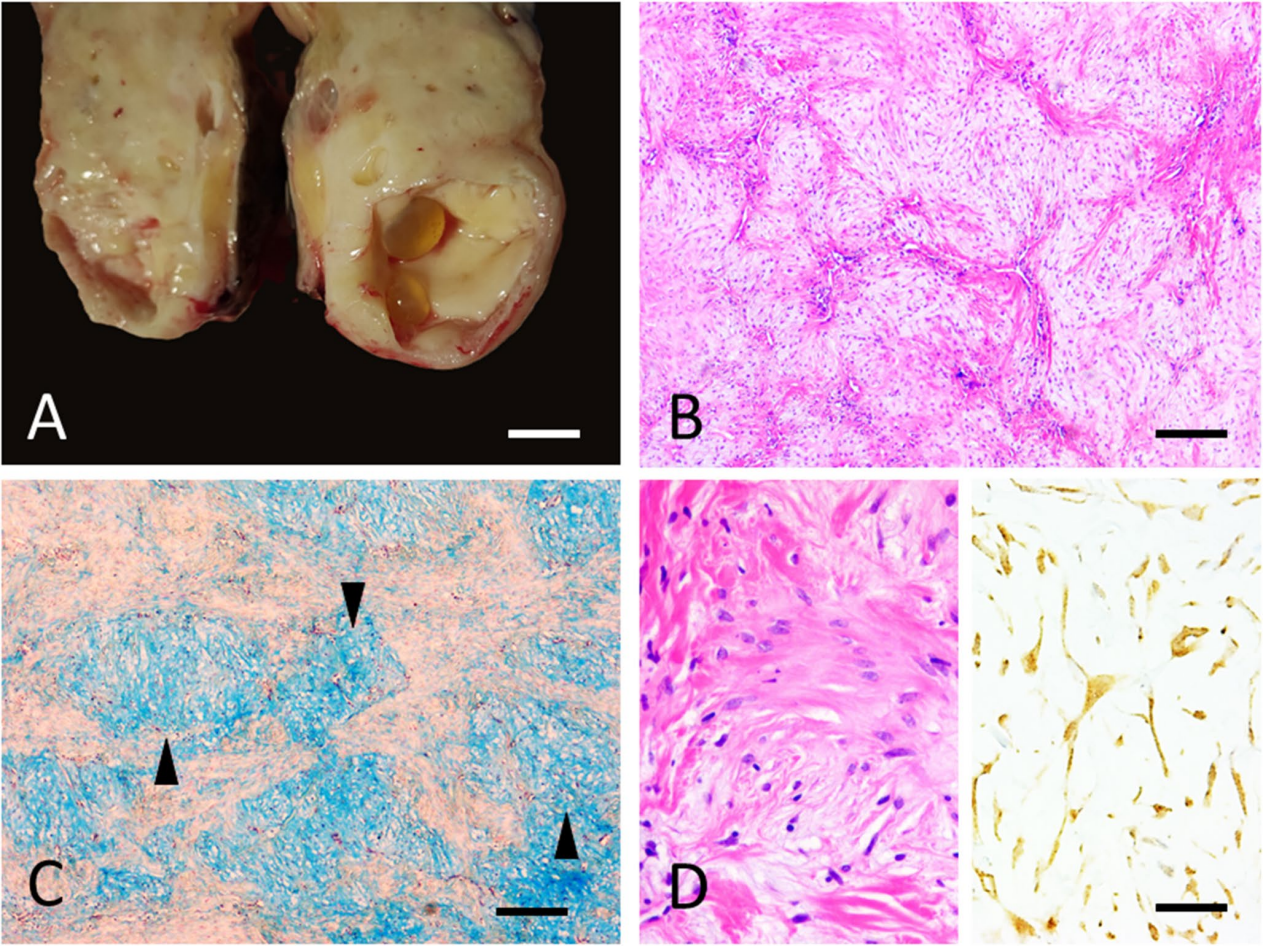
Resume of gross and microscopic findings of multiple cutaneous Nerve Sheath Tumors of farmed Russian sturgeons. **A** Gross features. The cutaneous masses on sectioning appear homogeneously whitish-grey and firm. Cystic spaces containing yellowish transparent fluid are interspersed with the solid masses (Bar = 1 cm). **B** The cutaneous masses are arranged in well-demarcated, moderately cellular, multilobular areas composed of spindle-to-stellate neoplastic cells organised in bundles (Haematoxylin and Eosin, bar = 500µ). **C** The neoplastic cells are intermixed with abundant extracellular alcianophilic material consistent with mucin (arrowheads) (Alcian Blue, bar = 500µ). **D** Left. Neoplastic spindle to stellate cells show moderate anisocytosis and anisokaryosis. Mitoses are less than one per high-power field. Right. Immunohistochemistry with the S-100 antibody show diffuse and marked cytoplasmic staining of neoplastic cells (Left and right images bar = 50µ)

With respect to pathological findings, the cutaneous masses were cauliflower-shaped and whitish; on sectioning, they appeared homogeneous with a pale, whitish-grey colour and firm consistency. Cystic spaces containing yellowish transparent fluid were observed interspersed with the solid masses (Fig. 1A). Other gross findings were unremarkable.

Histologically, the cutaneous masses affecting the dermis were arranged in well-demarcated, expansile, moderately cellular, multilobular areas composed of spindle-to-stellate neoplastic cells organised in bundles (Fig. 1B). The neoplastic cells were loosely arranged and intermixed with optically empty or pale eosinophilic, amorphous, alcianophilic material consistent with mucin (Fig. 1C). Multifocally, the neoplastic cells exhibited a stellate appearance and were PAS-positive. Anisocytosis and anisokaryosis were moderate. Mitoses were less than one per high-power field (Fig. 1D, left). Mild, multifocal lymphoplasmacytic inflammation was present at the periphery of the neoplastic tissue.

IHC with the S-100 antibody showed diffuse and marked cytoplasmic specific staining of neoplastic cells (Fig. 1D, right). Based on these findings, a diagnosis of cutaneous nerve sheath tumor (NST) with myxoid differentiation was made.

## Discussion

The literature describing cutaneous masses in sturgeons is very limited, and tumours in these species have not been documented to date. Johnston et al. (2022) reported skin masses in adult wild sturgeons from the Great Lakes (*Acipenser fulvescens*, Rafinesque 1877) that histologically resembled hyperplastic epidermitis associated with herpesvirus-like virions. In another study involving 15 farmed Russian sturgeons, cauliflower-like masses on the skin and in the oral cavity were diagnosed as mycobacterial granulomas (Antuofermo et al. 2014).

Cutaneous masses of neoplastic origin have been reported in other farmed fish species. These include myxomas in California yellowtail (*Seriola lalandi*, Valenciennes 1833) (Keller et al. 2011), and NSTs in lake trout (*Salvelinus namaycush*, Walbaum 1792) (Spitsbergen et al. 2013) and rainbow trout (*Oncorhynchus mykiss*, Walbaum 1792) (Brocca et al. 2021). Additionally, multicentric nerve sheath myxoma was described in a population of farmed European eels (*Anguilla anguilla*, Linnaeus 1758) (Gurcevic et al. 2014). The authors of that report proposed the term “myxoma” based on the histopathological findings, which were characterised by abundant loose tissue forming the ground substance, and the absence of reliable immunohistochemical results.

Regarding the possible causes of dermal proliferation in European eels, it was hypothesised that water temperature changes during early developmental stages, combined with genetic predisposition, may have contributed to tumour induction. We are of the opinion that some morphologic similarities exist between the above-mentioned tumours in eels and the Russian sturgeons in the present study, particularly concerning the morphology of neoplastic cells and the type of ground substance. Further parallels between the two cases may relate to the aquatic environment, which could act as a non-specific but concurrent predisposing factor for disease.

In addition, a report on *Acipenser gueldenstaedtii* (Brandt and Ratzeburg 1833) and *Acipenser baeri* (Brandt 1869) noted the occasional presence of tumours weighing up to 3 kg at the base of the ventral fins in fish older than 2 years (Kayiş et al. 2017), although histological confirmation of these findings was not provided.

Regarding the potential tumour origin of the current cases, and as a general consideration, a genetic aetiology could also be considered. However, such evidence is very difficult to establish. Although certain fish species exhibit predispositions to naturally occurring neoplasms, the tendency of some species to develop particular types of tumours may relate more to habitat than to genetics (Frasca et al. 2018).

In investigating the cause of the masses in the sturgeons described in this report, a viral origin was considered. However, analyses targeting the most common agents associated with sturgeon diseases, Acipenser iridovirus and Acipenser herpesvirus (Bondavalli et al. 2024; Ciulli et al. 2016; Johnston et al. 2022), did not identify any aetiological agents. This negative result was further supported by the absence of viral particles on ultrastructural examination.

Given the localisation of the cutaneous masses in highly exposed regions of the sturgeons, which frequently come into contact with the substrate and tank walls, repeated mechanical trauma might provide a plausible explanation for the tissue proliferation observed. Sparse literature has suggested a potential link between sarcoma development and trauma (Montgomery et al. 2019).

## Conclusions

The cutaneous masses observed in the sturgeons described in this report exhibit common macroscopic and histologic features. Based on these findings, and to the best of our knowledge, this represents the first description of multiple NSTs with myxoid differentiation in this species. Morphological similarities were noted between the neoplastic cells in these cases and those of cutaneous myxomas in European eels and NST in rainbow trout. The potential causes, including factors intrinsic to the fish and influences from the aquatic environment, have been discussed. Since similar morphological and immunohistochemical features were noted among previously described cutaneous tumors in European eels, a rainbow trout, a lake trout and our cases it would be interesting to investigate further the mechanisms behind the development of NSTs in fish since this histotype appears to be relatively common in these species.

## Data Availability

No datasets were generated or analysed during the current study

## References

[CR1] Antuofermo E, Pais S, Nuvoli S, Hetzel U, Burrai GP, Rocca S, Caffara M, Giorgi I, Pedron C, Prearo M (2014) *Mycobacterium chelonae* associated with tumor-like skin and oral masses in farmed Russian sturgeons (*Acipenser gueldenstaedtii*). BMC Vet Res 10:18. 10.1186/1746-6148-10-18 24423126 10.1186/1746-6148-10-18 PMC3898693

[CR2] Bigarré L, Lesne M, Lautraite A, Chesneau V, Leroux A, Jamin M, Boitard PM, Toffan A, Prearo M, Labrut S, Daniel P (2017) Molecular identification of iridoviruses infecting various sturgeon species in Europe. J Fish Dis 40:105–118. 10.1111/jfd.12498 27193445 10.1111/jfd.12498

[CR3] Bondavalli F, Schleicherová D, Pastorino P, Mugetti D, Pedron C, Prearo M (2024) Detection of Acipenser European Iridovirus (AcIV-E) in Sturgeon Farms in Northern Italy between 2021–2023. Viruses 16:465. 10.3390/v16030465 38543830 10.3390/v16030465 PMC10975281

[CR4] Brocca G, Zamparo S, Quaglio F, Verin R (2021) Metastatic myxoid nerve sheath tumor of the dorsal fin in a rainbow trout, *Oncorhynchus mykiss* (Walbaum). J Fish Dis 44:1875–1878. 10.1111/jfd.13517 34432897 10.1111/jfd.13517

[CR5] Bronzi P, Chebanov M, Michaels JT, Wei Q, Rosenthal H, Gessner J (2019) Sturgeon meat and caviar production: global update 2017. J Appl Ichthyol 35:257–266. 10.1111/jai.13870

[CR6] Ciulli S, Volpe E, Sirri R, Passalacqua PL, Cesa Bianchi F, Serratore P, Mandrioli L (2016) Outbreak of mortality in Russian (*Acipenser gueldenstaedtii*) and siberian (*Acipenser baerii*) sturgeons associated with sturgeon nucleo-cytoplasmatic large DNA virus. Vet Microbiol 191:27–34. 10.1016/j.vetmic.2016.05.012 27374904 10.1016/j.vetmic.2016.05.012

[CR7] Elhetawy AIG, Vasilyeva LM, Lotfy AM, Emelianova NA, Abdel-Rahim MM, Helal AM, Sudakova N (2020) Effects of the rearing system of the Russian sturgeon (*Acipenser gueldenstaedtii*) on growth, maturity, and the quality of produced caviar. AACL Bioflux 13:6. http://www.bioflux.com.ro/aacl

[CR8] Frasca S Jr, Wolf JC, Kinsel MJ, Camus AC, Lombardini ED (2018) Chapter 39. Osteichthyes. In: Terio K, Mc Aloose D, St. Leger J (eds) Pathology of Wildlife and Zoo Animals, 1st edition. Academic Press, pp 953–1001

[CR9] Gurcevic E, Kužir S, Sfacteria A, Drašner K, Marino F (2014) Spontaneous multicentric myxoma of the dermal nerve sheaths in farmed European eels *Anguilla anguilla*. DAO 111:173–176. 10.3354/dao02746 10.3354/dao02746 25266905

[CR10] Hanson LA, Rudis MR, Vasquez-Lee M, Montgomery RD (2006) A broadly applicable method to characterize large DNA viruses and adenoviruses based on the DNA polymerase gene. Virol J 11:28–38. 10.1186/1743-422X-3-28 10.1186/1743-422X-3-28 PMC1459111 16608526

[CR11] Hedrick RP, McDowell TS, Groff JM, Yun S, Wingfield WH (1991) Isolation of an epitheliotropic herpesvirus from white sturgeon Acipenser transmontanus. Dis Aquat Organ 11:49–56. 10.3354/dao011049

[CR12] Johnston AE, Shavalier MA, Scribner KT, Soto E, Griffin MJ, Waldbieser GC, Richardson BM, Winters AD, Yun S, Baker EA et al (2022) First isolation of a Herpesvirus (Family Alloherpesviridae) from Great Lakes Lake Sturgeon (*Acipenser fulvescens*). Animals 12:3230. 10.3390/ani12233230 36496751 10.3390/ani12233230 PMC9740441

[CR13] Kayis Ş, Er A, Kangel P, Kurtoğlu İZ (2017) Bacterial pathogens and health problems of *Acipenser gueldenstaedtii* and *Acipenser baerii* sturgeons reared in the eastern Black Sea region of Turkey. Iran J Vet Res Winter 18:18–24 PMC5447134 28580011

[CR14] Keller M, Han S, Snekvik K (2011) Severe anisakiasis and cutaneous myxoma in a California yellowtail, *Seriola lalandi* Valenciennes. J Fish Dis 34:635–639. 10.1111/j.1365-2761.2011.01276x 21762175 10.1111/j.1365-2761.2011.01276.x

[CR15] Montgomery C, Park KJ, Gardner JM, Majors I, Nicholas R (2019) Post-traumatic sarcomas: do they exist? Int J Surg Pathol 27:722–728. 10.1177/1066896919848495 31208254 10.1177/1066896919848495

[CR16] Mugetti D, Pastorino P, Menconi V, Pedron C, Prearo M (2020) The Old and the New on viral diseases in Sturgeon. Pathogens 21(2):146. 10.3390/pathogens9020146 10.3390/pathogens9020146 PMC7168591 32098100

[CR17] Spitsbergen JM, Frattini SA, Bowser PR, Getchell RG, Coffee LL, Wolfe MJ, Fisher JP, Marinovic SJ, Harr KE (2013) Epizootic neoplasia of the lateral line system of lake trout (*Salvelinus namaycush*) in New York’s Finger Lakes. Vet Pathol 50:418–433. 10.1177/0300985813482949 23528941 10.1177/0300985813482949

[CR18] Telenti A, Marchesi F, Balz M, Bally F, Böttger EC, Bodmer T (1993) Rapid identification of mycobacteria to the species level by polymerase chain reaction and restriction enzyme analysis. J Clin Microbiol 31:175–178 8381805 10.1128/jcm.31.2.175-178.1993 PMC262730

[CR19] Vilkova D, Chèné C, Kondratenko E, Karoui RA (2022) A comprehensive review on the assessment of the quality and authenticity of the sturgeon species by different analytical techniques. Food Control 133:108479. 10.1016/j.foodcont.2021.108648

